# Dry‐Milled Corn and Corn Products and Health: A Narrative Review

**DOI:** 10.1002/fsn3.71518

**Published:** 2026-02-13

**Authors:** Mark Kern, Shirin Hooshmand

**Affiliations:** ^1^ School of Exercise and Nutritional Sciences San Diego State University San Diego California USA

**Keywords:** corn, dry‐milled, health, maize, nutrients

## Abstract

Corn (maize) is commonly consumed after dry‐milling. Common products include whole or refined grits, cornmeal, corn flour, and more that can produce foods including polenta, tortillas, and tortilla chips, corn chips, breakfast cereals, and more. Processing can also include nixtamalization, which introduces alkali to the product. Whole grain products tend to be richer in nutrients that are lost when the bran and germ are removed to produce refined products. Epidemiological research supports the consumption of whole dry‐milled corn products. Limited clinical trials tend to report positive results for whole grain products and mixed results for refined products. Critical needs exist for long‐term dose–response studies of both whole and refined foods. Dry‐milled products should be studied for potential impacts on macular health via zeaxanthin. The potential values that dry‐milled corn products such as corn and tortilla chips, as well as breakfast cereal, as carriers for healthful foods should be explored.

## Graphical abstract

This review describes research regarding products of dry‐milled corn (maize) including potential health implications. Foods can be produced from whole or more highly refined versions of grits, cornmeal, or corn flour and may or may not be nixtamalized, which can impact nutrient contents and bioavailability. Whole grain products tend to promote health while refined products yield mixed results. Future directions should include long‐term trials, evaluation as a carrier food and health impacts of phytochemicals present.

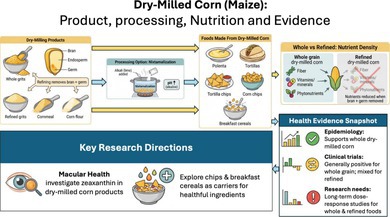

## 1 Introduction

**FIGURE 1:**
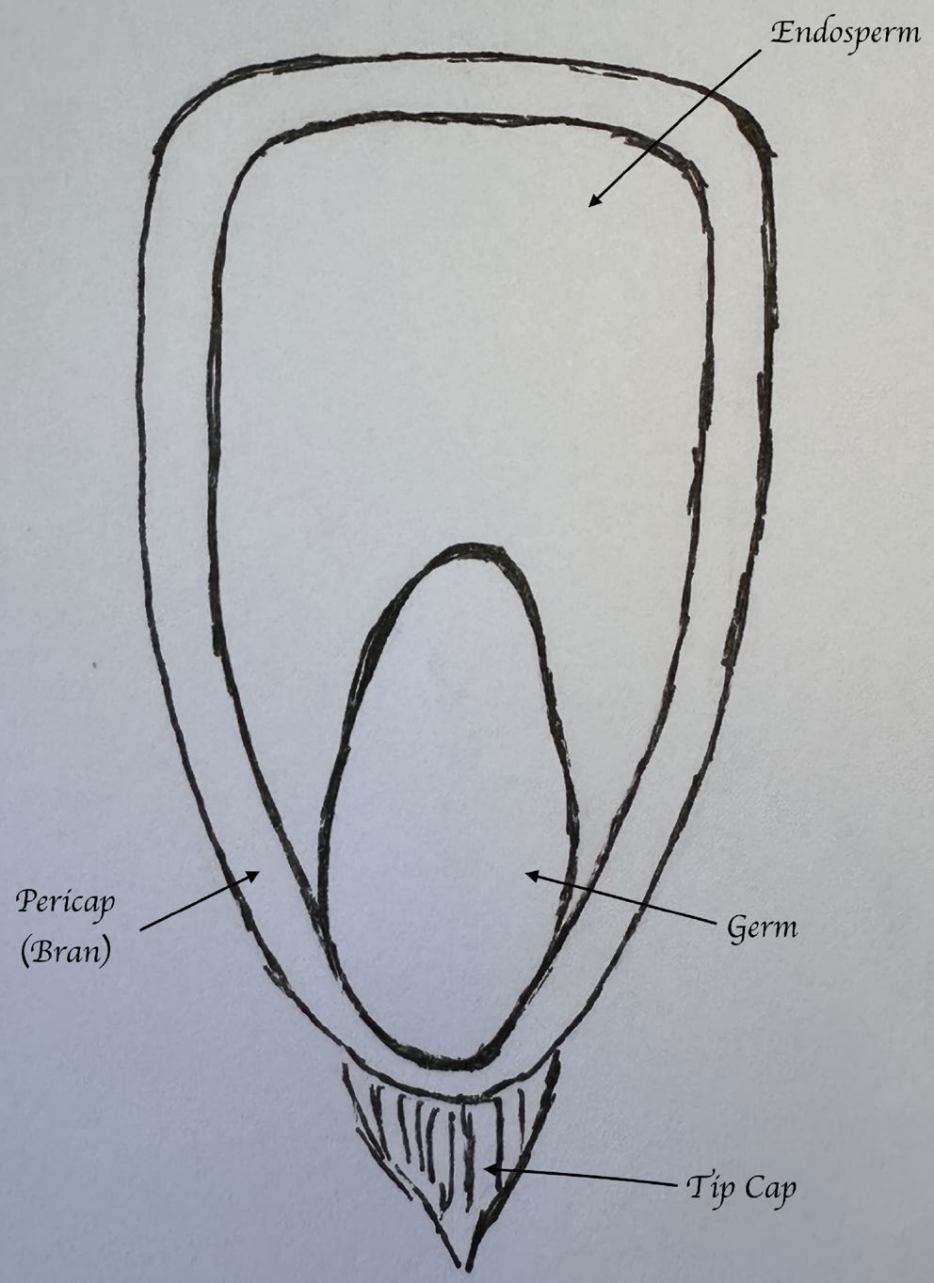
Components of a corn kernel.

Corn ( *Zea mays* L), also known as maize, has served as a food source for humans for thousands of years. Corn is commonly eaten as whole kernels; however, much of the corn and corn products consumed today has been milled. Milling processes can generally be divided into dry‐milling and wet‐milling. Dry‐milling is a mechanical process that separates corn kernels into distinct components, primarily the endosperm, germ, and bran/pericarp. Dry‐milling processes are summarized as depicted in Figure 1. The complexities of production of various products derived from dry‐milling have been previously detailed and are beyond the scope of this review (Serna‐Saldivar et al. 2000; Gwirtz and Garcia‐Casal 2014).

Dry‐milled corn and corn products are widely consumed and include both whole grain foods as well as refined grains, primarily including dry‐milled products that have been degermed. Preparations of dry‐milled corn include white, yellow, and blue varieties of corn meals, corn grits, corn flour, and pregelatinized corn flour, which can be used in a wide array of food products. Other products of dry‐milling such as corn bran can be used as an ingredient in numerous corn products. Popcorn is also a dried corn product but is a product of the whole kernel rather than a product of the milling process.

Despite the widespread consumption of dry‐milled corn and corn products, surprisingly little research has been conducted to determine how these foods influence health. Plate and Gallaher (2005) reviewed the potential health benefits of corn components and products. However, that review included discussions of fresh corn products in addition to products of the dry‐milling process, which they concluded had up to that point received little research attention, particularly beyond the healthful corn bran and corn fiber fractions. They also highlighted the possibility for some corn products to serve as a key source of antioxidants and called for more research regarding the potential for corn products to impact the development of chronic diseases. More recently, Mohr and Whisner (2025) published a scoping review regarding the impacts of corn flour on health effects throughout life; however, that review was largely confined to relatively narrow analyses of 21 studies that fit within their review criteria. The major conclusions drawn by those authors were that the long‐term impacts of processed corn products and extruded snacks remain controversial. Among their findings were that no association has been detected for colorectal cancer risk, but that a higher risk of obesity has been reported; that in limited research fasting triglycerides may be elevated by these foods, but that typically no adverse effects on blood lipids are reported; that corn bran and whole corn consumption tend to improve blood lipids; and that better blood glucose responses have been observed with consumption of corn fiber, tortillas made to be rich in resistant starch, and corn flour that is high in amylose. The goal of this review is to describe corn products of the dry‐milling process and to examine the literature regarding the health outcomes of consuming dry‐milled corn and corn products. Secondary aims are to identify gaps in the scientific literature and provide suggestions for types of research that might fill those gaps.

## 2 Components of the Corn Kernel

**FIGURE 2:**
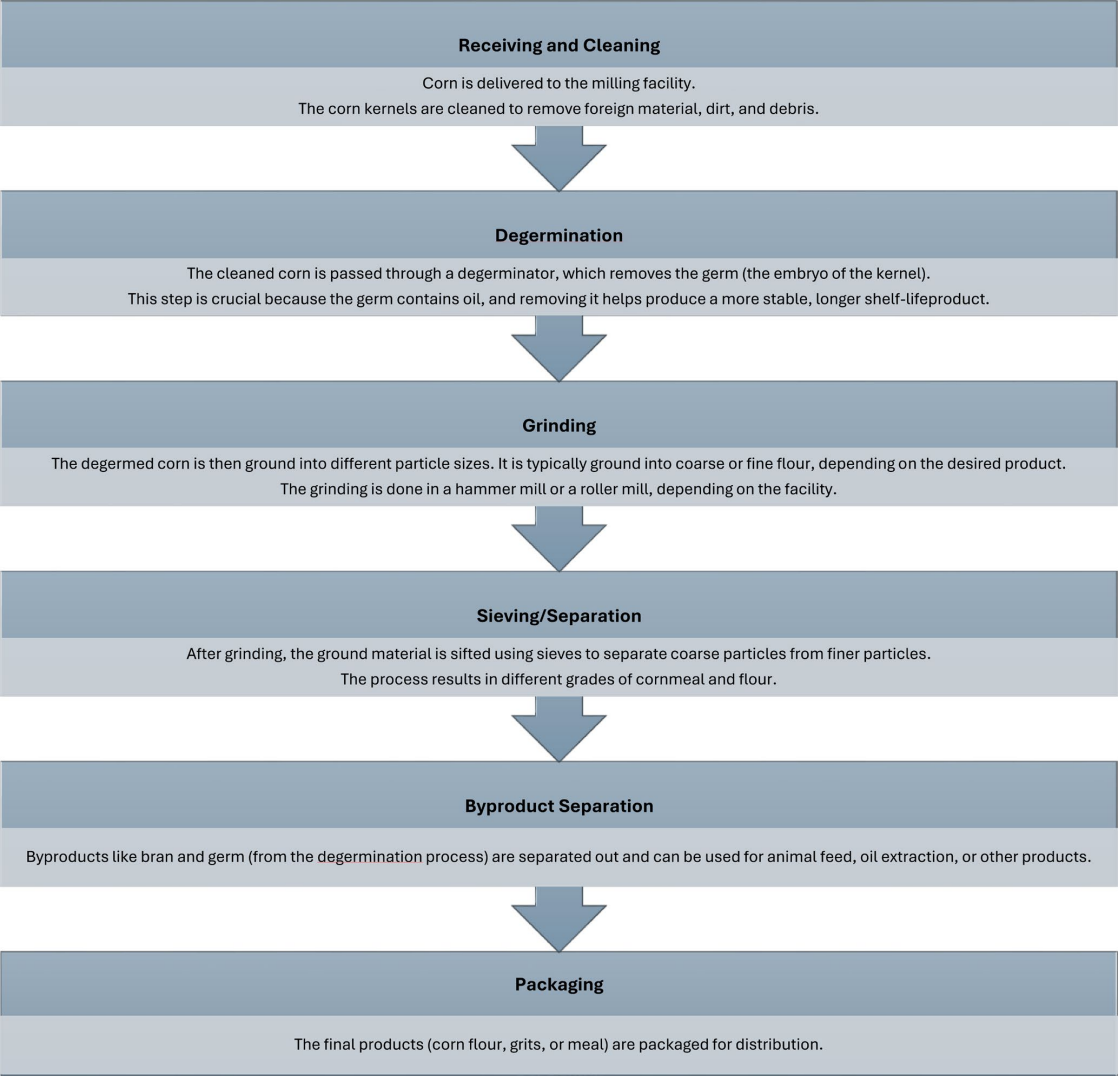
Dry‐milling of corn and production of dry‐milled corn products.

Key components (Figure 2) of the corn kernel include the endosperm, germ, bran/pericarp, and tipcap, which have been described in detail (Serna‐Saldivar et al. 2000). The endosperm represents the largest fraction of the kernel at approximately 82% by dry weight. This component primarily consists of starch molecules, but also includes some protein and is a minor provider of other nutrients. Its main purpose is to serve as an energy source for young plants during germination. In food, the endosperm is a major component of products such as cornmeal, grits, and flour. The germ is the seed embryo and typically accounts for 11%–12% of the kernel's weight. The germ is the major site of key nutrients of corn, including fat, which is primarily composed of polyunsaturated fatty acids, vitamins (especially vitamin E but also numerous other vitamins including several B vitamins), minerals, and some protein. The germ remains present in whole grain dry‐milled products but is often absent in more refined foods. When present, the germ serves as a rich source of nutrients; however, it is often removed for product development to promote a longer shelf life. The outer covering of the kernel is known as the bran, which includes the pericarp that serves to protect the kernel from pests, disease, and loss of moisture. This component is approximately 5%–6% of the weight of the kernel and is the component that contains the majority of the corn fiber as well as some antioxidant compounds including ferulic acid. Like the germ, this component is often removed during processing but is present in whole grain corn products and can be used as an ingredient in foods to enhance nutrient composition. The tip cap is a minor component of the kernel and serves as a small attachment point to the corn cob. It consists mostly of fibrous material and acts to channel water and nutrients to the kernel via the cob.

## 3 Products Made From Dry‐Milled Corn

Dry‐milled corn and corn products are used in the production of a variety of food products. Cornmeal, which is ground corn, is a common product from dry‐milling and can be found in foods as both whole grain cornmeal containing both the bran and germ and as refined cornmeal in which the bran and germ are generally absent. Foods produced from cornmeals include cornbread, muffins, tortillas, and others. More coarsely ground corn results in what is known as corn grits, which is particularly common in Southern‐style cooking of the US. Likewise, polenta, often considered a component of Italian cuisine but consumed in many regions, is a cornmeal‐based food that is produced by boiling cornmeal. When cornmeal is more finely ground, it results in the production of corn flour, which is particularly useful for baking and as a coating for frying foods. Corn flour can be obtained as either whole (such as masa harina) or degermed varieties without the bran as well. When cornmeal is more highly refined, the extracted starch from the endosperm can be used for producing cornstarch, which is commonly used as a thickener for foods such as soups, sauces, gravies, and puddings. However, much of the cornstarch on the market is also obtained from wet‐milling processes rather than dry‐milling; therefore, studies of cornstarch were generally not included in this review.

Some foods are produced through the additional processing of nixtamalization as described by Serna‐Saldivar et al. (2000). This process includes the soaking and cooking of the corn in an alkali such as lime. The process is particularly common in countries of Mesoamerica and is used for making masa, which commonly includes removal of the pericarp. Commercially, much of the masa is dried to produce varieties of masa harina (dry masa flour) that are commonly used to make tortillas, tortilla chips, and tamales.

In their comprehensive review, Gwirtz and Garcia‐Casal (2014) provide a further description of globally consumed foods derived from dry‐milled corn. Among breads, they indicate that both flat, unleavened and unfermented products include tortillas and arepas. Fermented and/or leavened varieties include blintzes, pancakes, corn bread, and hoe cake. They classify foods such as tamales, couscous, rice‐like products, Chinese breads, dumplings, and chengu that are made from dry‐milled corn as steamed products. They indicate that both fermented and unfermented porridges are commonly consumed in some countries. Lastly, they feature the following as snack foods produced from dry‐milled corn: empanadas, chips, tostadas, popped corn, and fritters. This extensive list of foods highlights the widespread consumption of numerous foods that often serve as staple foods in various parts of the world and further emphasizes the importance of gaining a more complete understanding of the nutritional value of these various foods.

## 4 Nutritional Profiles of Dry‐Milled Corn Products

The nutritional profiles of corn and corn products depend upon the extent of processing. Table 1 provides a summary of the nutritional profiles of various dry‐milled corn products using information from the United States Department of Agriculture's Food Data Central website. As can be seen, some differences exist based on the degree of refinement as well as whether or not the product has been refined. Only slight differences are evident among corn varieties of different color. Notably, perusal of the table reveals significant overlap of the data among several products, which calls into question the veracity of all of the data presented. The table indicates that whole grain corn varieties tend to be richer in key nutrients such as fat, fiber, and key micronutrients including vitamins and minerals in comparison to refined corn that has been degermed and dehulled. Likewise, enriched products are higher in thiamin, riboflavin, niacin, and folate than the unenriched counterparts.

**TABLE 1:** Nutritional composition of key products of dry‐milling of corn expressed as units per 100 g of product.

Name	Unit	Yellow cornmeal (Navajo)	White cornmeal	Blue cornmeal	Degermed unenriched white cornmeal	Degermed enriched white cornmeal	Degermed unenriched yellow cornmeal	Degermed enriched yellow cornmeal	Corn flour yellow whole grain	Corn flour yellow degermed unenriched	Corn flour yellow masa enriched	Corn grits yellow unenriched	Corn bran
Water	g	10.2	5.42	5.7	11.2	11.2	11.2	11.2	10.9	9.81	9.79	10	4.71
Energy	kcal	384	398	398	370	370	370	370	361	375	363	371	224
Energy	kJ	1610	1660	1670	1550	1550	1550	1550	1510	1570	1520	1550	937
Protein	g	9.85	11	10.4	7.11	7.11	7.11	7.11	6.93	5.59	8.46	8.8	8.36
Total lipid (fat)	g	5.88	5.04	5.44	1.75	1.75	1.75	1.75	3.86	1.39	3.69	1.2	0.92
Ash	g	1.22	1.4	1.54	0.51	0.51	0.51	0.51	1.45	0.46	1.48	0.4	0.36
Carbohydrate	g	72.9	77.1	76.9	79.4	79.4	79.4	79.4	76.8	82.8	76.6	79.6	85.6
Fiber, total dietary	g	9.4	10.4	8.7	3.9	3.9	3.9	3.9	7.3	1.9	6.4	1.6	79
Total Sugars	g	1.56	1.46	1.81	1.61	1.61	1.61	1.61	0.64	0.64	NA	0.64	0
Sucrose	g	1.2	1.16	1.53	0.68	0.68	0.68	0.68	NA	NA	NA	NA	NA
Glucose	g	0.22	0.18	0.16	0.56	0.56	0.56	0.56	NA	NA	NA	NA	NA
Fructose	g	0.15	0.11	0.12	0.17	0.17	0.17	0.17	NA	NA	NA	NA	NA
Maltose	g	0	0	0	0.19	0.19	0.19	0.19	NA	NA	NA	NA	NA
Starch	g	61.9	60.5	63.6	73.3	73.3	73.3	73.3	NA	NA	66	NA	NA
Calcium, Ca	mg	6	11	5	3	3	3	3	7	2	138	2	42
Iron, Fe	mg	2.99	3.79	2.91	1.1	4.36	1.1	4.36	2.38	0.91	8.5	1	2.79
Magnesium, Mg	mg	107	125	133	32	32	32	32	93	18	93	27	64
Phosphorus, P	mg	225	280	354	99	99	99	99	272	60	231	73	72
Potassium, K	mg	322	443	393	142	142	142	142	315	90	262	137	44
Sodium, Na	mg	4	4	7	7	7	7	7	5	1	5	1	7
Zinc, Zn	mg	3.1	3.24	2.91	0.66	0.66	0.66	0.66	1.73	0.37	1.8	0.41	1.56
Copper, Cu	mg	0.242	0.219	0.218	0.076	0.076	0.076	0.076	0.23	0.142	0.209	0.075	0.248
Manganese, Mn	mg	0.641	0.646	0.758	0.174	0.174	0.174	0.174	0.46	0.056	0.376	0.106	0.14
Selenium, Se	μg	6	NA	11.8	10.5	10.5	10.5	10.5	15.4	8	10.5	17	16.5
Vitamin C	mg	0	0	0	0	0	0	0	0	0	0	0	0
Thiamin	mg	0.3	0.31	0.285	0.14	0.551	0.14	0.551	0.246	0.074	1.48	0.13	0.01
Riboflavin	mg	0.093	0.137	0.107	0.05	0.382	0.05	0.382	0.08	0.058	0.805	0.04	0.1
Niacin	mg	2.47	2.8	2.02	1	4.97	1	4.97	1.9	2.66	9.93	1.2	2.74
Pantothenic acid	mg	0.595	2.49	0.353	0.24	0.24	0.24	0.24	0.658	0.052	0.192	0.485	0.636
Vitamin B‐6	mg	0.59	0.583	0.593	0.182	0.182	0.182	0.182	0.37	0.097	0.475	0.147	0.152
Folate, total	μg	34	32	58	30	209	30	209	25	48	162	5	4
Folic acid	μg	0	0	NA	0	180	0	180	0	0	133	0	0
Folate, food	μg	34	32	58	30	30	30	30	25	48	29	5	4
Folate, DFE	μg	34	32	NA	30	335	30	335	25	48	256	5	4
Alpha‐tocopherol	mg	0.37	0.37	0.73	0.12	0.12	0.12	0.12	0.42	0.15	NA	NA	0.58
Tocopherol, beta	mg	0	0	0	0.02	0.02	0.02	0.02	NA	NA	NA	NA	NA
Tocopherol, gamma	mg	4.86	5.06	3.21	0.45	0.45	0.45	0.45	NA	NA	NA	NA	NA
Tocopherol, delta	mg	0.37	0.52	0.37	0.04	0.04	0.04	0.04	NA	NA	NA	NA	NA
Tocotrienol, alpha	mg	0.37	0.58	0.37	0.35	0.35	0.35	0.35	NA	NA	NA	NA	NA
Tocotrienol, beta	mg	0	0	0	0	0	0	0	NA	NA	NA	NA	NA
Tocotrienol, gamma	mg	0.99	1.08	0.55	0.58	0.58	0.58	0.58	NA	NA	NA	NA	NA
Phylloquinone	μg	0.2	0.4	0	0	0	0	0	0.3	0.3	NA	0.3	0.3
Dihydrophylloquinone	μg	0	0.6	0	0	0	0	0	NA	NA	NA	NA	NA
Vitamin B‐12	μg	NA	NA	NA	0	0	0	0	0	0	NA	NA	0
Vitamin A, RAE	μg	11	NA	NA	11	0	11	11	11	11	11	11	4
Vitamin A, IU	IU	NA	NA	NA	214	3	214	214	214	214	214	214	71
Carotene, beta	μg	NA	NA	NA	97	1	97	97	97	97	97	97	32
Carotene, alpha	μg	NA	NA	NA	63	0	63	63	63	63	63	63	21
Cryptoxanthin, beta	μg	NA	NA	NA	0	1	0	0	0	0	0	0	0
Lutein + zeaxanthin	μg	1359	NA	NA	1630	5	1630	1630	1360	1360	6	1360	1360
Choline, total	mg	21.6	NA	NA	8.6	8.6	8.6	8.6	21.6	NA	NA	NA	18.1
Betaine	mg	11.6	NA	NA	1	1	1	1	NA	NA	NA	NA	4.6
Fatty acids, total saturated	g	1.04	0.853	0.886	0.22	0.22	0.22	0.22	0.543	0.171	0.532	0.155	0.13
SFA 8:0	g	0	0	0	0	0	0	0	0	0	NA	0	0
SFA 10:0	g	0	0	0	0	0	0	0	0	0	NA	0	0
SFA 12:0	g	0	0	0	0.001	0.001	0.001	0.001	0	0.001	NA	0.001	0
SFA 14:0	g	0	0	0	0.001	0.001	0.001	0.001	0	0.001	NA	0.001	0
SFA 16:0	g	0.778	0.671	0.713	0.175	0.175	0.175	0.175	0.463	0.14	0.453	0.135	0.111
SFA 18:0	g	0.219	0.133	0.132	0.038	0.038	0.038	0.038	0.061	0.025	0.06	0.018	0.015
SFA 20:0	g	0.033	0.029	0.025	0.004	0.004	0.004	0.004	NA	NA	NA	NA	NA
SFA 22:0	g	0.013	0.02	0.016	0	0	0	0	NA	NA	NA	NA	NA
Fatty acids, total MUFA	g	2.14	1.53	1.68	0.39	0.39	0.39	0.39	1.02	0.274	0.997	0.3	0.243
MUFA 16:1	g	0	0	0.003	0.003	0.003	0.003	0.003	0.003	0.004	0.003	0.004	0.001
MUFA 18:1	g	2.13	1.52	1.66	0.386	0.386	0.386	0.386	1.02	0.274	0.994	0.296	0.243
MUFA 20:1	g	0.017	0.017	0.015	0	0	0	0	0	0	NA	0	0
Fatty acids, total PUFA	g	2.35	2.08	2.46	0.828	0.828	0.828	0.828	1.76	0.695	1.72	0.516	0.421
PUFA 18:2	g	2.29	2.02	2.4	0.808	0.808	0.808	0.808	1.71	0.673	1.67	0.502	0.408
PUFA 18:3	g	0.06	0.054	0	0.02	0.02	0.02	0.02	0.053	0.022	0.052	0.015	0.013
PUFA 18:3 n‐3 c, c, c (ALA)	g	0.06	0.054	0.061	NA	NA	NA	NA	NA	NA	NA	NA	NA
Tryptophan	g	0.05	0.07	NA	0.038	0.038	0.038	0.038	0.049	NA	0.062	0.062	NA
Threonine	g	0.307	0.345	NA	0.172	0.172	0.172	0.172	0.261	NA	0.245	0.33	NA
Isoleucine	g	0.37	0.404	NA	0.242	0.242	0.242	0.242	0.248	NA	0.276	0.314	NA
Leucine	g	1.28	1.38	NA	1.01	1.01	1.01	1.01	0.85	NA	1.02	1.08	NA
Lysine	g	0.301	0.319	NA	0.105	0.105	0.105	0.105	0.195	NA	0.219	0.247	NA
Methionine	g	0.23	0.258	NA	0.162	0.162	0.162	0.162	0.145	NA	0.167	0.184	NA
Cystine	g	0.184	0.217	NA	0.159	0.159	0.159	0.159	0.125	NA	0.182	0.158	NA
Phenylalanine	g	0.499	0.543	NA	0.366	0.366	0.366	0.366	0.34	NA	0.411	0.431	NA
Tyrosine	g	0.294	0.412	NA	0.187	0.187	0.187	0.187	0.282	NA	0.229	0.357	NA
Valine	g	0.494	0.554	NA	0.337	0.337	0.337	0.337	0.351	NA	0.38	0.444	NA
Arginine	g	0.421	0.474	NA	0.239	0.239	0.239	0.239	0.345	NA	0.375	0.437	NA
Histidine	g	0.265	0.292	NA	0.172	0.172	0.172	0.172	0.211	NA	0.26	0.268	NA
Alanine	g	0.769	0.836	NA	0.56	0.56	0.56	0.56	0.518	NA	0.635	0.656	NA
Aspartic acid	g	0.662	0.724	NA	0.465	0.465	0.465	0.465	0.482	NA	0.546	0.61	NA
Glutamic acid	g	1.88	2.04	NA	1.46	1.46	1.46	1.46	1.3	NA	1.58	1.65	NA
Glycine	g	0.36	0.399	NA	0.217	0.217	0.217	0.217	0.284	NA	0.313	0.36	NA
Proline	g	0.854	0.906	NA	0.746	0.746	0.746	0.746	0.605	NA	0.676	0.765	NA
Serine	g	0.471	0.51	NA	0.341	0.341	0.341	0.341	0.329	NA	0.416	0.417	NA

Protein represents approximately 9.85%–11% protein by dry weight among various varieties. The protein fraction of corn is typically low in lysine and tryptophan. In contrast, corn is rich in the sulfur‐containing amino acids methionine and cysteine. While corn is not typically considered a “complete protein,” this amino acid profile allows the protein quality to easily extend the protein quality of most animal foods and to be complemented by various plant foods such as legumes. A prime example of protein complementation is milpa farming (also known as the “three sisters” method of farming corn, beans, and squash), which is a traditional farming practice in regions of the Americas.

Conversely, corn bran obtained from dry‐milling can serve as a rich source of fiber and phytonutrients, which may be particularly beneficial to the cells of the colon and can serve as prebiotics for microorganisms residing there. Specific fractions of dry‐milled corn can also be obtained. Notably, soluble corn fiber is used to enrich various foods, and data are accumulating as to its nutritional benefits (Whisner et al. 2016), which is covered in more detail later.

Nutritional profiles of other preparations of corn products including enriched and unenriched masa flour and precooked corn flours are described by Gwirtz and Garcia‐Casal (2014). Masa flour tends to be higher in certain nutrients, such as calcium that is added during nixtamalization, as well as thiamin, riboflavin, and niacin and lower in folate.

Zeaxanthin is a key bioactive component of dry‐milled corn. Although dietary composition tables such as Food Data Central from the USDA often include the sum of the carotenoids lutein and zeaxanthin contents of foods within their tables, zeaxanthin is primarily consumed from either corn products or eggs. With regard to eggs, the zeaxanthin tends to originate from corn fed to hens, which explains its presence in eggs since laying hens are often fed dried corn. The term zeaxanthin is derived from the Greek root zea, which refers to corn or maize, and xanthose, which means yellow. Zeaxanthin appears to possess important biological activity. Notably, lutein and zeaxanthin are the two carotenoids present in the macula of the eye. As has been previously reviewed, their presence apparently protects the macula from oxidative damage that can result in macular loss as occurs with macular degeneration (Li et al. 2023). Comprehensive analyses of zeaxanthin levels in snack foods and breakfast cereals produced from dry‐milled corn are not available. Furthermore, the contribution of dry‐milled corn products to overall zeaxanthin status deserves investigation.

Among grain foods, corn is a particularly rich source of ferulic acid (Della Pepa et al. 2018), which is largely confined to the bran component of the corn kernel (Mathew and Abraham 2004). Plate and Gallaher (2005) reviewed research regarding corn as a provider of ferulic acid. They concluded that overall studies point toward corn as among the grains with the highest total antioxidant capacity, which is at least partially due to the high ferulic acid content. The authors make a strong case for the capacity of ferulic acid from dry‐milled corn to have beneficial effects for nitric oxide production, colon cancer prevention, decreased protein and lipid oxidation, blood pressure lowering, and improved glycemic control; however, research regarding the bioavailability of ferulic acid from corn products (muffins and bread) suggests that absorption by intestinal cells is likely poor (Abdel‐Aal et al. 2023), perhaps because ferulic acid is largely bound to arabinoxylan in corn. These data suggest that ferulic acid from dry‐milled corn may exert its greatest effects in the colon where it is likely released and could be available to the colonic cells or the gut microbiota (Kroon et al. 1997).

As reviewed by Žilić et al. (2022), snack chips made from corn have also been demonstrated to contain acrylamides. A recent comprehensive review suggests that acrylamides have been linked to aberrant health outcomes that may increase disease risk when consumed at high doses (Govindaraju et al. 2024), although more research is needed since some rich sources of acrylamide such as coffee, dark chocolate, and roasted nuts and seeds have been linked to health benefits indicating that acrylamide does not always pose a danger. Methods such as nixtamalization with lime or the addition of calcium chloride or magnesium chloride in low concentrations or nixtamalization have been proposed to mitigate acrylamide formation in processed corn foods (Arámbula‐Villa et al. 2018; Topete‐Betancourt et al. 2019). These methodologies should be considered by the snack chip industry as a way to lessen the exposure of the public to these potentially harmful compounds. Development of a database of acrylamide levels in commercially available snack foods would be useful for researchers and practitioners and potentially for consumers as well.

Table 2 provides an interpretive synthesis contrasting typical whole‐grain versus refined dry‐milled corn products. These general patterns are modified by nixtamalization, enrichment/fortification, added ingredients (fat, sugar, sodium), and preparation method. Given their ubiquitous nature in the US food supply, specific nutritional issues of snack foods and breakfast cereals produced from dry‐milled corn should be considered.

**TABLE 2:** Whole‐grain versus refined dry‐milled corn products: typical compositional and functional differences.

Feature	Whole‐grain dry‐milled corn products (bran + germ retained)	Refined dry‐milled corn products (degermed/dehulled; largely endosperm)
Kernel fractions present	Endosperm, germ, and bran/pericarp are retained (or largely retained)	Primarily endosperm; germ and bran largely removed
Dietary fiber & food matrix	Higher fiber and a more intact matrix; resistant‐starch formation can occur after cooking/cooling depending on product	Lower fiber; finer particle size and extrusion can increase rapidly available starch
Lipids & vitamin E	Higher unsaturated fat and vitamin E due to germ retention (shorter shelf‐life if not stabilized)	Lower fat and vitamin E; typically longer shelf‐life
Micronutrients	Generally higher minerals and some B vitamins; levels vary by variety and processing	Lower natural micronutrients; some products are enriched/fortified (e.g., B vitamins/iron)
Phytochemicals	More phenolics (e.g., ferulic acid concentrated in bran) and typically more carotenoids when pigmented varieties are used	Reduced bran‐associated phenolics; carotenoids may be lower depending on fraction/variety
Nixtamalization considerations	Can increase calcium and alter fiber/phenolics depending on pericarp removal and formulation	Similar considerations; refined masa/flour products may have added calcium yet limited bran‐associated components
Typical food applications	Whole‐grain cornmeal/polenta, some whole‐grain tortillas/tortilla chips, products with added corn bran/fiber	Degermed cornmeal/grits/flour, many snack chips and flaked/puffed cereals, cornstarch‐based foods
Interpretation in this review	When epidemiology or trials specify whole‐grain corn/whole‐corn products, findings are most directly relevant	Findings on refined corn foods often reflect formulation (fat/sodium/sugar) and processing, not corn per se

*Note:* The characteristics above are general patterns; helps interpret sections where corn is included within broader grain categories but not analyzed independently.

### 4.1 Snack Foods and Breakfast Cereals Made With Dry‐Milled Corn

Dry‐milled corn ingredients are widely used in snack foods and ready‐to‐eat breakfast cereals. Their nutrient profiles are highly heterogeneous; the most important driver is whether products are made from whole‐grain fractions (bran and germ retained) versus refined fractions (degermed/dehulled). Independent of grain fraction, preparation method (baked vs. fried or extruded), added oils/fats, sodium, and added sugars can substantially influence energy density and overall dietary quality.

### 4.2 Snack Foods (Chips and Extruded Snacks)

Tortilla chips, corn chips, and extruded corn snacks are frequently produced from refined dry‐milled corn, although whole‐grain formulations exist. Compared with refined versions, whole‐grain formulations generally provide more dietary fiber, vitamin E, minerals, and bran‐associated phytochemicals. Across both types, frying/baking conditions, the oil source, and seasoning levels (particularly sodium) are key determinants of nutrient density and cardiometabolic relevance. Because these foods are commonly consumed with accompaniments (e.g., vegetable/legume dips, avocado‐based dips, or dairy‐based toppings), their potential role as carrier foods for nutrient‐dense additions deserves explicit study.

### 4.3 Breakfast Cereals

Corn‐based breakfast cereals (e.g., flaked or puffed products) commonly use refined corn ingredients and are often fortified with selected micronutrients; added sugars vary widely by product. Therefore, when interpreting evidence on corn‐based cereals, it is important to distinguish whole‐grain versus refined formulations and to account for added sugars and fortification. Like snack foods, breakfast cereals may function as carrier foods for other food groups, particularly dairy and fruit, which may modify net health impacts.

While knowledge of the nutritional profiles of dry‐milled corn has grown, more complete and accurate information is needed. Such information would help to inform researchers for the development of studies designed to assess the nutritional implications of these products.

## 5 Potential Health Implications of Dry‐Milled Corn and Corn Products

Research regarding the potential health implications of dry‐milled corn and corn products is scant, particularly given their common consumption in a variety of commercially available products as described. The available studies can be broken down into categories of both epidemiological studies and experimental research that include a small number of clinical trials. Furthermore, studies with both categories of research include those of whole grain dry‐milled foods, refined varieties of those foods, as well as functional ingredients such as bran and corn fiber produced through the dry‐milling process.

### 5.1 Epidemiological Research

Epidemiological evidence specific to dry‐milled corn products is limited. Most observational studies either (a) include corn/maize within broad whole‐grain exposure variables without isolating corn, or (b) examine corn‐based foods that are typically refined and/or highly processed (e.g., extruded snacks or flaked cereals). To improve interpretability, the evidence is summarized below by level of corn specificity and by degree of refinement.

#### 5.1.1 Whole‐Grain Literature That Includes Corn/Maize (Corn Not Isolated)

Several large reviews and primary studies define whole grains broadly and may include corn/maize among many grains, but rarely report grain‐specific estimates. For example, a dose–response meta‐analysis of prospective cohorts reported lower all‐cause and cardiometabolic mortality risk with higher whole‐grain intake, noting inconsistencies in whole‐grain definitions and the specific foods captured across cohorts (Benisi‐Kohansal et al. 2016). Similarly, cross‐sectional analyses that define grains to include wheat, rice, corn, oats, and grain‐based foods generally find inverse associations between total whole‐grain intake and cardiometabolic risk factors and less favorable profiles with refined‐grain intake, with cereal fiber implicated as a key contributor (Newby et al. 2007). These findings support whole‐grain consumption broadly, but they should not be interpreted as corn‐specific effects when corn is not analyzed independently.

#### 5.1.2 Studies Evaluating Corn/Maize or Corn‐Based Staples Separately

A smaller set of observational studies provides corn/maize‐specific information. In the Danish Diet, Cancer and Health cohort, total whole‐grain intake was associated with lower myocardial infarction risk; however, in grain‐specific analyses, rye and oats (but not wheat, corn/maize, or other grains) were significantly associated with lower risk (Helnæs et al. 2016). In Venezuelan adults, arepa consumption (a refined corn‐flour staple) was inversely associated with prevalence of obesity, hypertension, and type 2 diabetes in a cross‐sectional analysis; interpretation is limited by potential confounding and reverse causality (Goodman et al. 2020).

#### 5.1.3 Refined Corn Snack/Cereal Products (Processed Foods)

Evidence specific to refined corn‐based snacks and cereals is mixed and generally comes from case–control or cross‐sectional designs. In Iranian children, more frequent consumption of extruded corn snacks was reported among those with obesity compared with nonobese controls (Baygi et al. 2013). In Jordanian adults, the frequency of cornflakes and extruded snacks was not associated with colorectal cancer in a case–control study (Tayyem et al. 2016). Studies of broader dietary patterns (e.g., snack patterns, deep‐fried foods) may include corn‐ or tortilla‐chip consumption (Wei et al. 2021, Zhong et al. 2022) but these designs make it difficult to attribute associations to dry‐milled corn ingredients per se.

Overall, the observational literature is consistent with benefits of whole grains as a category, yet provides limited capacity to isolate effects of dry‐milled corn products specifically. This limitation reinforces the need for well‐designed trials that compare whole‐grain versus refined dry‐milled corn foods under controlled conditions and in relevant populations.

### 5.2 Experimental Studies

#### 5.2.1 Acute Metabolic Responses

The majority of studies assessing the metabolic impacts of consuming whole grain corn/corn products have measured the glycemic responses to ingestion of these foods, often in comparison to more refined counterparts. The available research was reviewed in 2005 by Plate and Gallaher who concluded that many foods produced from dry‐milled corn such as corn chips, popcorn, hominy, and tortillas exhibit low glycemic indexes and exert low glycemic loads. Those authors also concluded that foods produced with corn products that have been milled to have smaller particle sizes raise the glycemic responses to a greater degree and that cornmeal that has been cooked and cooled yields a lower glycemic response, presumably through the formation of resistant starch. The more recent review of Mohr and Whisner (2025) came to similar conclusions.

Breakfast cereals are a common application of dry‐milled corn, but relatively few trials evaluate these foods beyond acute postprandial responses. The available studies largely involve refined corn‐based cereals (e.g., cornflakes) compared with higher‐fiber cereals (e.g., bran‐based flakes or oats). Across these comparisons, differences in satiety and gastric emptying are generally consistent with differences in fiber content and degree of processing rather than corn as a grain per se (Hlebowicz et al. 2007; Geliebter et al. 2015). Evidence comparing corn grits with other groats similarly suggests high satiety overall, with stronger effects for higher‐fiber groats such as oats and barley (Skotnicka et al. 2018).

Taken together, the available data suggest that whole grain corn products tend to promote favorable impacts on acute glycemic responses and that impacts are sometimes but not always better for whole corn vs. refined corn products. Since both fresh whole corn kernels as well as dry‐milled whole cornmeal have low glycemic indexes, the degree of processing and the addition of other ingredients such as higher glycemic sugars are likely responsible for higher glycemic indexes of some dry‐milled corn‐based foods. Limitation of some of those ingredients in commercial products may yield foods that exhibit a more healthful response, which would be particularly helpful for less healthy populations.

#### 5.2.2 Longer‐Term Intervention Studies

Some researchers have conducted longer‐term interventions. For example, researchers at the University of Reading, England carried out a 3‐week double‐blind, placebo‐controlled, cross over human feeding study to assess the impacts of consuming a whole maize (corn) cereal daily in 32 healthy adults (Carvalho‐Wells et al. 2010). The researchers provided either 48 g of whole grain cereal produced from dry‐milled corn or a placebo cereal without whole grain corn. Assessment of the gut microbiome revealed increases in the abundance of potentially beneficial bifidobacteria levels after eating the whole grain corn in comparison to the refined corn product. Although no changes in blood lipid profiles or glucose concentrations were detected, this research further highlights the potential of whole grain corn products to promote human health. A short‐term study indicated that despite similar glucose responses during a glucose tolerance test at the end of 2 weeks, swapping a muesli cereal for cornflakes produced a lower insulinemic response and higher daylong glucose concentrations (Golay et al. 1992). The authors suggested that individuals with diabetes might rely on less exogenous insulin treatment by simply replacing more highly refined cornflakes with muesli. In similar research, Geliebter et al. (2014) compared the responses to consuming frosted cornflakes to oat porridge as well as to a water only trial for 4 weeks. The oat porridge promoted greater fullness and less hunger throughout the 4 weeks in comparison to the cornflakes. Both of those groups exhibited no change in blood lipids, body composition, insulin response or blood pressure; whereas, total cholesterol was elevated within the control group. Moreover, among overweight individuals, 50 g of available carbohydrate from a barley‐based cereal has been demonstrated to promote better metabolic and satiety outcomes in comparison to cornflake cereal during a 6‐week intervention (Kang et al. 2025). Most significantly, the barley cereal group exhibited more weight loss and lower glycated albumin at the end of the trial. In contrast, the corn cereal group gained approximately 0.85 kg, which may have occurred secondary to a diminished acute GLP‐1 and hunger responses and/or the fact that the barley feeding provided approximately 3 g more fiber than the cornflake feeding.

Postive results of that study are reinforced by those of Liedike et al. (2024) who recently assessed the impacts of 4 weeks of consumption of 48 g per day of whole‐grain cornmeal compared to refined cornmeal as well as to a blend of refined cornmeal and corn bran incorporated into muffins and pita bread on cardiometabolic outcomes and the gut microbiome. Key outcomes included a significant lower of LDL‐cholesterol by the blend of refined cornmeal with added corn bran as well as an increase in the *Agathobaculum* genus by the whole‐grain cornmeal. Overall, this study suggests that whole‐grain components of corn may modestly improve health outcomes in comparison to more highly refined cornmeal. Notably, the muffins and pita breads were well accepted regardless of formulation, and no differences in gastrointestinal symptoms after consumption of the various products were detected.

Although snack chips produced from dry‐milled corn products are commonly consumed, their impacts on health outcomes in randomized trials have not been well studied. However, researchers have evaluated the impacts of feeding a high fat diet providing 12%–15% of energy from corn and tortilla snack chips fried in corn oil for 25 days on risk factors for cardiovascular disease (St‐Onge et al. 2007). The diet containing the snack chips lowered LDL‐cholesterol and total cholesterol concentrations more so than a high fat diet with less polyunsaturated fatty acids and than a lower fat diet and tended to lower triglyceride concentrations. That study suggests that at least when fried in a polyunsaturated fat such as corn oil, corn and tortilla chips do not inherently produce aberrant health effects contrary to popular notions.

Aside from measurements of the glycemic indexes of various dry‐milled corn products and the few studies of other outcomes reviewed above, surprisingly little research has attempted to specifically assess the impacts of whole or refined corn products obtained through dry‐milling on health outcomes. The studies that are available provide a mixed picture demonstrating that much more research is needed since these foods are widely consumed.

## 6 Functional Ingredients From Dry‐Milled Corn

### 6.1 Soluble Corn Fiber

In recent years, the potential functional impact of soluble corn fiber has gained attention. For example, Tan et al. (2020) examined the impact of glucose beverages consumed prior to four meals that provided soluble corn fiber or maltodextrin. Inclusion of soluble corn fiber in the meals lowered the glucose and insulin responses of the participants. This research followed a study of Konings et al. (2014) that demonstrated the capacity for soluble corn fiber to lower glucose and insulin response and to promote fat oxidation.

Furthermore, soluble corn fiber has been demonstrated to promote positive changes in the gut microbiome that were associated with enhanced absorption of dietary calcium (Whisner et al. 2016). This study suggests that the addition of soluble corn fiber to foods may ultimately lead to improvements in bone health.

### 6.2 Corn Starch

Although the majority of industrially‐produced corn starch is produced through wet‐milling procedures, many dry‐milled products retain the starch component of corn, which makes consideration of the health consequences of starch consumption relevant to this review. To date, relatively few nutrition‐related studies have evaluated the impacts of corn starch on human health; however, perhaps surprisingly, the available data point toward potential health benefits rather than detriments. For example, Swanson et al. (1992) utilized foods containing corn starch (primarily corn flour) along with starch from bread, other sources of wheat, potatoes, and oats as a comparator component in research designed to assess the effects of fructose on metabolic outcomes. In that research, neither fructose nor starch consumed at 20% of energy intake yielded negative effects on lipid profiles or blood glucose after 28 days of consumption. Whether similar results would have been obtained if 100% corn starch had been fed is unknown.

## 7 Future Research

Given the ubiquity of dry‐milled corn product ingredients and foods within the food supply, a more complete understanding of their impacts on health outcomes is critical. Several areas for further investigation have been mentioned throughout the manuscript. Key suggestions for research include the assessment of the dose response of a variety of commercially available dry‐milled corn foods as whole grains and both nixtamalized and non‐nixtamalized refined foods using both pre‐clinical and clinical designs. This research should be conducted in a variety of both healthy and less healthy populations of various ages, sexes, and physical activity levels to determine how well tolerated and accepted both whole grain and refined grain products are. These studies would be particularly valuable given the current concern regarding consumption of “ultraprocessed foods.” Furthermore, many of the available snack foods, cereals, and other foods represent the opportunity to evaluate their potential impacts as carrier foods on health outcomes. With regard to snack foods such as corn/tortilla chips, the potential for these foods to promote consumption of potentially healthful foods such as avocados (particularly served as guacamole), vegetable/bean dips, and melted cheese represents a valuable avenue of research. Likewise, the capacity of breakfast cereals produced from either whole or refined dry‐milled corn as a carrier food for dairy products and fruits should be studied to maximize potential health outcomes of consumers. Data are emerging regarding the value of zeaxanthin for macular health; therefore, the capacity of this nutrient to improve macular health when consumed as part of a diet rich in dry‐milled corn, perhaps focusing on whole grain products, should be considered.

Furthermore, since the Dietary Guidelines for Americans recommends that at least half of grain consumption be from whole grains, it is imperative to determine if this guideline is supported by empirical data for whole and refined dry‐milled corn products. If research determines that indeed half of these grain foods can be supplied by refined products, the message that there is no need for completely avoiding these foods in their refined forms. Such research could lessen the burden of consumers with regard to their selections of foods containing these products. Furthermore, this could represent an opportunity for food manufacturers to develop and market more products that contain a mix of whole and refined dry‐milled corn ingredients in ways that can optimize palatability while nourishing consumers. Furthermore, researchers of behavioral nutrition should consider assessing the psychosocial value of inclusion of refined dry‐milled corn products in the diet.

Another potential avenue for research is the development and efficacy of both whole and refined dry‐milled corn ingredients in sports foods. Given the need for higher levels of energy consumption among some athletes as well as the particularly high carbohydrate contents of these foods, a wide variety of sports foods in various forms could be produced.

Overall, future research should determine if consumer perceptions of products made with whole grains influence acceptability. When possible, the use of whole grain corn for acceptable products should be encouraged by both practitioners and industry. In the absence of such research, a major goal for the future should be to conduct high quality, physiologically relevant human trials of typical portion sizes of commonly consumed products to determine the impacts of dry‐milled corn products on health outcomes. Furthermore, while the Dietary Guidelines for Americans call for consumers to consume at least half of grains as whole grains, this recommendation has not been critically evaluated by empirical randomized controlled studies. This oversight in nutrition research should be rectified given the prevalence of foods in the US food supply produced from dry‐milled corn.

## 8 Conclusion

Overall, accumulating data suggest that whole grain dry‐milled corn and corn products may promote positive health outcomes. Furthermore, some research indicates that more refined dry‐milled corn foods may have some positive and some negative health consequences. Since dry‐milled corn is widely consumed, much research is warranted to understand the dietary roles for both whole and refined dry‐milled corn foods. With regard to current recommendations for consumption, based on the limited data that are available, we suggest that consumers consider inclusion of a variety of whole grain corn products in their diets when possible. We further suggest that healthy individuals can incorporate modest amounts of refined corn products including both snack foods, particularly those that are baked or fried in healthful oils, and breakfast cereals; however, we recommend that individuals with known chronic diseases or risk factors for chronic diseases limit their consumption and enjoy those foods as carrier foods for healthful ingredients.

## Author Contributions

**Mark Kern:** conceptualization; investigation; writing – original draft; project administration; funding acquisition. **Shirin Hooshmand:** conceptualization; investigation; writing – review and editing; illustrations; project administration; funding acquisition. Both authors approve the final version of the manuscript and agree to be accountable for all aspects of the work.

## Funding

This work was supported by the Grain Foods Research Institute.

## Conflicts of Interest

The authors declare no conflicts of interest.

## Data Availability

The data that support the findings of this study are available on request from the corresponding author. The data are not publicly available due to privacy or ethical restrictions.
